# Time dependence of evolutionary metrics during the 2009 pandemic influenza virus outbreak

**DOI:** 10.1093/ve/vev006

**Published:** 2015-08-24

**Authors:** Austin G. Meyer, Stephanie J. Spielman, Trevor Bedford, Claus O. Wilke

**Affiliations:** ^1^Department of Integrative Biology, Institute for Cellular and Molecular Biology, and Center for Computational Biology and Bioinformatics, The University of Texas at Austin, Austin, TX, USA, 78712; ^2^School of Medicine, Texas Tech University Health Sciences Center, Lubbock, TX, USA, 79430; ^3^Vaccine and Infectious Disease Division, Fred Hutchinson Cancer Research Center, Seattle, WA, USA, 98109

**Keywords:** influenza, molecular clock rate, *dN*/*dS*, emerging infectious diseases, evolution

## Abstract

With the expansion of DNA sequencing technology, quantifying evolution during emerging viral outbreaks has become an important tool for scientists and public health officials. Although it is known that the degree of sequence divergence significantly affects the calculation of evolutionary metrics in viral outbreaks, the extent and duration of this effect during an actual outbreak remains unclear. We have analyzed how limited divergence time during an early viral outbreak affects the accuracy of molecular evolutionary metrics. Using sequence data from the first 25 months of the 2009 pandemic H1N1 (pH1N1) outbreak, we calculated each of three different standard evolutionary metrics—molecular clock rate (i.e., evolutionary rate), whole-gene *dN*/*dS*, and site-wise *dN*/*dS*—for hemagglutinin and neuraminidase, using increasingly longer time windows, from 1 month to 25 months. For the molecular clock rate, we found that at least 3–4 months of temporal divergence from the start of sampling was required to make precise estimates that also agreed with long-term values. For whole-gene *dN*/*dS*, we found that at least 2 months of data were required to generate precise estimates, but 6–9 months were required for estimates to approach their long term values. For site-wise *dN*/*dS* estimates, we found that at least 6 months of sampling divergence was required before the majority of sites had at least one mutation and were thus evolutionarily informative. Furthermore, 8 months of sampling divergence was required before the site-wise estimates appropriately reflected the distribution of values expected from known protein-structure-based evolutionary pressure in influenza. In summary, we found that evolutionary metrics calculated from gene sequence data in early outbreaks should be expected to deviate from their long-term estimates for at least several months after the initial emergence and sequencing of the virus.

## 1 Introduction

While modern medicine has been able to control the prevalence of traditional human pathogens through vaccines and medications, emerging infectious diseases remain a major threat to public health. Many emerging infectious diseases are caused by zoonotic viruses, which are normally endemic to a reservoir animal and are transmitted to humans upon exposure to the respective reservoir. Some viruses that have successfully undergone zoonoses into humans during the last century include HIV, Zaire ebolavirus, Hantavirus, Machupo virus, Marburg virus, and Chickungya virus, as well as most major influenza virus subtypes, including pandemic 1918 H1N1, H2N2, H3N2, pH1N1, H5N1, and the recent H7N9.

Quantifying the evolutionary dynamics of emerging zoonotic epidemics has become an important tool in understanding and controlling such epidemics. Computational methods that extract evolutionary information from gene-sequence data can produce valuable hypotheses regarding viral adaptation (Bush et al. 1999b; Luksza and Lassig 2014). Two particular metrics have been widely used to analyze such evolutionary dynamics from genetic data: the molecular clock rate (also known as the clock rate, the nucleotide substitution rate, or the evolutionary rate) and the evolutionary rate ratio, *dN*/*dS* (Bush et al. 1999a; Rambaut and Holmes 2009; Bhatt et al. 2013). The molecular clock rate indicates the rate of sequence divergence, whereas the *dN*/*dS* rate ratio gives the relative rate of non-synonymous to synonymous sequence changes and is widely used to infer positive selection (when dN/dS>1). In viral sequences, dN/dS>1 is thought to reflect viral adaptation, typically in response to selection pressure exerted by the host immune system. Most commonly, the clock rate is measured on an entire genome or occasionally on a single gene, and *dN*/*dS* is measured either on a gene or at individual sites in a gene.

Importantly, each of these metrics is computed using models of molecular evolution which assume that observed mutations represent fixed population differences, not intra-population polymorphisms. This assumption has wide-reaching consequences for inferences. A growing body of theoretical work has demonstrated that data collected over short-time scales may yield biased estimates for both the molecular clock rate (Ho et al. 2005, 2007; Peterson and Masel 2009; Ho et al. 2011; Biek et al. 2015) and *dN*/*dS* (Rocha et al. 2006; Kryazhimskiy and Plotkin 2008; dos Reis and Yang 2013; Mugal, Wolf, and Kaj 2014). Moreover, a pattern of over-estimation in the molecular clock rate relative to long-term estimates has been shown in a small number of real viral systems (Wertheim and Kosakovsky-Pond 2011). This would be expected either because sufficient time has not passed for natural selection to purge slightly deleterious (often non-synonymous) mutations from the gene pool or because not enough mutations have accumulated to correctly compute a *dN*/*dS* ratio. Much of this work has additionally demonstrated that, after sequences have sufficiently diverged, the clock rate and *dN*/*dS* estimates do converge to a long-term steady-state value (Ho et al. 2007; Peterson and Masel 2009; Mugal, Wolf, and Kaj 2014). We therefore expect that evolutionary estimates computed with sequences from early outbreak stages will not be reliable approximations of longer term values. It is not known, however, how much time is required to obtain long-term clock rates or *dN*/*dS* estimates either per gene or in the site-wise distribution.

Here, we have investigated the extent to which limited sampling divergence time produces molecular clock rate and *dN*//*dS* estimates in an emerging virus that do not agree with long-term estimates for that virus. Specifically, we have analyzed the hemagglutinin (pH1) and neuraminidase (pN1) sequences from the 2009 pandemic H1N1 (pH1N1) to systematically examine how divergence time influences clock rate and *dN*/*dS* estimates both across the entire protein and at each site individually. Previously, Hedge, Lycett, and Rambaut (2013) conducted a similar time-series study of pH1N1. Their analysis included an estimate of R_0_, an analysis of the time dependence in the molecular clock rate, and the identification of the most recent common ancestor with whole-genome data. We have performed a more detailed analysis of the two genes that dominate influenza antigenicity and have included calculations of the gene-wise molecular clock rate, the whole-gene *dN*/*dS*, and the site-wise *dN*/*dS* (Bush et al. 1999a; Luksza and Lassig 2014; Meyer and Wilke 2015). Thus, the two studies are complementary for quantifying the evolution of pH1N1.

We have found that, early in the outbreak, both clock rate and *dN*/*dS* estimates are not equal to their long-term, steady-state values. In particular, when only the first month of sequence data is used to generate estimates, the clock rate is three to five times higher than the value obtained after 25 months of divergence in the sample. Similarly, we have found that whole-gene *dN*/*dS* for pH1 and pN1 are approximately 30 per cent higher for pH1 and 50 per cent lower for pN1 than their values after 25 months. Additionally, the majority of site-wise *dN*/*dS* estimates are completely uninformative until at least 6 months of mutations have accumulated. Finally, we have found that at least 8 months of accumulated sample divergence are required for site-wise *dN*/*dS* calculations to reflect structural constraints on protein evolution.

Taken together, our results indicate that some of the most commonly used metrics in molecular evolution can be very different in early outbreaks relative to long-term estimates. In addition, considering the relatively rapid evolution of influenza, it is likely that most other emerging viruses will require substantially more divergence time than reported here for influenza. Therefore, investigators should expect that early estimates of the clock rate, the whole-gene *dN*/*dS*, and the site-wise *dN*/*dS* will likely not agree with their long-term values. Specifically, the early molecular clock rate will probably be elevated, the early whole-gene *dN*/*dS* may be unpredictably increased or decreased, and the early site-wise *dN*/*dS* will likely be uninformative in emerging outbreaks.

## 2 Materials and methods

### 2.1 Data collection and processing

All data analyzed were taken from the Influenza Research Database (IRD) (Squires et al. 2012). We specifically selected pH1N1 sequences for the genes hemagglutinin (pH1) and neuraminidase (pN1) collected from humans in North America, beginning in April 2009. We used the built-in IRD filter to include only pH1N1 sequences. Further, we used the built-in IRD filters to select specifically the pandemic strains. We downloaded sequences sampled from April 2009 to April 2011. We chose to exclude data dated after April 2011 because the number of available sequences began dropping quickly as pH1N1 became a seasonal strain, peaking in the winter and dropping to almost undetectable levels in the summer. We selected only sequences that represented the entire coding regions of the pH1 and pN1 genes. All laboratory strains, duplicate strains, and sequences which contained ambiguous nucleotides were omitted.

### 2.2 Molecular clock rate estimation

For molecular-clock rate estimation, we grouped sequences for each gene into time-aggregated datasets, as follows. We established a dataset for April 2009, a dataset for April–May 2009, a dataset for April–June 2009, and so on, until all months were included. Because datasets containing more than 2 months of data were too large for the estimation procedure to be computationally tractable, we down-sampled the datasets, as follows: For estimates in the third month, we aggregated twenty-five sequences from the first month, twenty-five sequences from the second month, and twenty-five sequences from the third month. Thus, estimates from June 2009 contained seventy-five sequences sampled at random from each month. For any month that did not have twenty-five sequences, we simply added all of the sequences from that month. For each subsequent time window, we added an additional twenty-five sequences from the next month (or all), such that the 25-month time window contained 470 sequences. For all subsampling, we used completely random sampling within each month’s sequences.

After compiling all datasets for all time windows, we converted each set of nucleotide sequences to amino acids and aligned them with MAFFT (Katoh and Standley 2013), specifying the ‘–auto’ flag. We then backtranslated each amino-acid alignment to the original codons.

For each alignment, we used BEAST (Drummond and Rambaut 2007) to infer the molecular clock rate. Here, a temporally dated phylogeny was estimated using BEAST with a logistic growth coalescent demographic model, an HKY nucleotide substitution model, and a strict molecular clock. The molecular clock rate was given a non-informative CTMC reference prior (Ferreira and Suchard 2008). Inclusion of gamma rate heterogeneity was avoided as preliminary analysis showed little effect on molecular clock rate with this data. Using a strict clock reduced the risk of model over-parameterization, as relaxed clock models add an additional parameter for every branch in the phylogeny, and strict clock models have been commonly used when studying influenza molecular evolution (Bedford et al. 2014). We ran Markov chain Monte Carlo (MCMC) for 500 million steps for most datasets and confirmed Bayesian convergence using Tracer. For the 25-month set, we ran MCMC until the runs had clearly converge via Tracer. All runs had an effective sample size in the 165–1,383 range.

To be sure that codon site heterogeneity was not affecting the molecular clock rate calculations, we repeated the MCMC calculation adding the gamma site heterogeneity model with four rate categories. The results were statistically identical to those we found previously (Supplementary Fig. S5). In addition, to ensure that sample divergence was increasing during the sampling period, we plotted the root height of the tree against the sampling time. We found a progressively taller phylogenetic tree as the sample time increased (Supplementary Fig. S6).

### 2.3 Estimating dN/dS for the whole-gene and individual sites

Estimation of *dN*/*dS* is commonly carried out by maximum likelihood, which scales to much larger dataset sizes than the Bayesian approach implemented in BEAST. Therefore, down-sampling of the data was not necessary for *dN*/*dS* estimates. Thus, for all *dN*/*dS* analyses, we established time-aggregated datasets as described in the previous subsection but did not down-sample. Again, we aligned each dataset by amino acids with MAFFT and then backtranslated to codons for subsequent *dN*/*dS* inference. We estimated a phylogeny for each alignment using FastTree v2.1.7 (Price, Dehal, and Arkin 2009), specifying the flags ‘-nt -gtr –nosupport’ to use the generalized time- reversible model.

Next, we used HyPhy (Kosakovsky Pond, Frost, and Muse 2005) to compute both whole-gene and site-wise *dN*/*dS* estimates for all alignments. We specifically used HyPhy’s MG94xHKY85 model (Kosakovsky Pond and Frost 2005; Kosakovsky Pond and Muse 2005), which is less biased than the more frequently used GY94 model (Spielman and Wilke 2015). In our estimates, we specified a single parameter *ω* to represent the *dN*/*dS* ratio, rather than using separate parameters for *dN* and *dS* as is often done in MG94 models (Kosakovsky Pond and Muse 2005). Site-specific *dN*/*dS* values were inferred using the fixed-effects likelihood method (Yang and Swanson 2002; Kosakovsky Pond and Frost 2005). As influenza proteins undergo substantial post-translational modification, we considered *dN*/*dS* values only for those sites which could be aligned to a known crystal structure, that is, sites which appeared in the mature protein. We obtained protein structures from the Protein Data Bank (Berman et al. 2000) with PDBID: 1RD8 for hemagglutinin and PDBID: 3TI3 for neuraminidase.

### 2.4 Calculating proximity to the receptor-binding site as a constraint on site-wise dN/dS

It has been shown that protein structure is a major determinant of hemagglutinin and neurminidase evolution (Meyer and Wilke 2013; Meyer, Dawson, and Wilke 2013; Sikosek and Chan 2014); in particular in hemagglutinin, distance to the sialic acid-binding site is a major constraint on hemagglutinin evolution (Meyer and Wilke 2015). For each time point, we evaluated the degree to which the distance from the sialic acid-binding site can predict the site-wise *dN*/*dS* of hemagglutinin.

To start, we first calculated the distances from every alpha–carbon to every other alpha–carbon. For hemagglutinin, this produced a square, symmetric matrix of 490 × 490 sites in the protein structure where the diagonal is all zeros (the diagonal represents the distance from a site to itself). To be clear, for hemagglutinin, the first column in the matrix (a 1 × 490 column) represents the distances from the first amino acid to all amino acids in the protein; the second column represents to distances from the second amino acid to all amino acids in the protein, etc. Then, for each column in the matrix, we calculated the correlation of site-wise *dN*/*dS* to the inverse distances for each site in the structure; again, for hemagglutinin, there were 490 *dN*/*dS* values (one for every site in the structure) and a set of 490 distances in each column (i.e., for each reference ${\text{C}}_{\alpha }$). We then plotted the correlation onto the structure at each amino acid site, using PyMOL (Schrödinger, LLC 2010). The same procedure was used to calculate distances using the neuraminidase structure and site-wise *dN*/*dS* values. As with hemagglutinin, we also used the inverse distance to compute correlations for neuraminidase.

We repeated this process for each month, where the site-wise *dN*/*dS* estimates were calculated with the aggregate of data for all previous months. Thus, at each time point, we were left with a distribution of correlations between distance and rates calculated with data up to that time point. The distribution of correlations was plotted as a violin plot for each time point (Figs 5 and 6); the violin plot should be seen as a horizontal, symmetric histogram. Thus, wider regions represent higher counts. For select time points (every 5 months), we plotted the distribution of correlations directly onto the protein structure below the violin plot. The figure shows that as more divergence was aggregated, the ability of this distance constraint to predict evolution in hemagglutinin and neuraminidase improved dramatically. This accompanies a characteristic flattening of the distribution of correlations; the flattening implies that as data are added, all of the sites in the protein appear to encode the same directional evolutionary pressure.

### 2.5 Statistical analysis and plotting

We used the R statistical programming language and the ggplot2 R package for all statistical analyses and plots, respectively (Ihaka and Gentleman 1996; Wickham 2009).

### 2.6 Availability

All code and data used are freely available from the github repository, https://github.com/wilkelab/influenza_pH1N1_timecourse.

## 3 Results

### 3.1 Brief history of pH1N1

The 2009 pandemic influenza (pH1N1) emerged in Mexico during March and April of 2009 and spread across the globe within weeks (Neumann, Noda, and Kawaoka 2009). Although the most common seasonal influenza strain in 2009 was H3N2, a descendant of the 1968 Hong Kong strain (Neumann, Noda, and Kawaoka 2009), pH1N1 accounted for as much as 99 per cent of the sub-typed, type A influenza infections after only a single season. The pH1N1 virus itself is thought to be a descendant of the 1998 triple re-assortment H3N2 strain after mixing with existing swine influenza strains (Neumann, Noda, and Kawaoka 2009; Smith et al. 2009; Vijaykrishna et al. 2011). Moreover, the two genes studied here, hemagglutinin and neuraminidase, descended from two separate influenza lineages (Smith et al. 2009). The pH1 hemagglutinin gene originated in the classical swine strain that diverged early last century from the human-infecting H1N1 virus, and the pN1 neuraminidase gene originated in the avian H1N1 lineage. Thus, pH1N1 represents a valuable study system for examining the evolution of two viral genes that had not adapted to humans for nearly 100 years, and this influenza strain is therefore an effective and representative model for a rapidly spreading infectious disease.

### 3.2 Over-estimation of molecular clock rate

**Figure 1.**
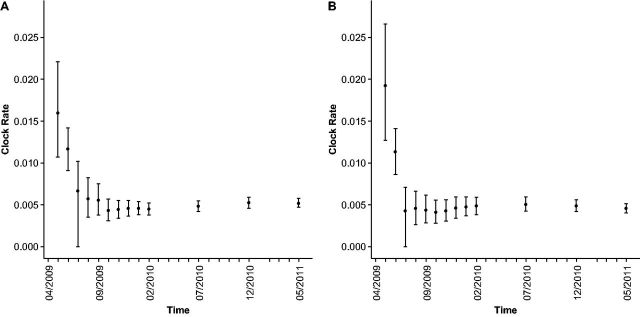
Molecular clock rate computed by BEAST for pH1 hemagglutinin and pN1 neuraminidase from the pH1N1 outbreak. In panel A, we show the molecular clock rate over time for pH1, and in panel B, we show the molecular clock rate over time for pN1. The error bars represent the HPD 95 per cent of the mean, as reported by BEAST. The plot shows a fourfold decline in the substitution rate estimates from single month of data to 25 months of aggregated data. Further, the molecular clock HPD 95 per cent for the first 2 months of data, for both pH1 and pN1, does not overlap the final clock rate, indicating that these early estimates are in no way representative of the long-term estimates

We calculated the molecular clock rate for pH1 and pN1 for each month in the outbreak from April 2009 to 2011, aggregating all available data up to each respective month (Fig. 1). We found that analyzing only the first month of data produced dramatically inflated molecular clock rates; the rate was overestimated roughly threefold for pH1 and fourfold for pN1. Upon adding the second month of data, the clock rate for pH1 declined substantially, and the pH1 clock rate converged to its long-term rate only after 5–6 months of data were incorporated. Similarly, for pN1, adding just 2–3 additional months of data to the first month produced molecular clock rate estimates that were essentially identical to the long term estimates. Thus, for influenza, molecular clock estimates could be computed with reasonable accuracy after approximately 4–6 months of sequence sample divergence. Note that for the remainder of this study, we will use the term ‘accuracy’ to refer to the extent to which any of the monthly estimates equal the long-term estimate. Similarly, we will use the terms ‘reliability’ or ‘precision’ to refer to the extent to which any of the estimates have low error of estimation.

Importantly, the 95 per cent highest probability density (HPD) intervals estimated 1–2 months after the start of the pandemic, although wide, did not include the long-term average (Fig. 1). Thus, the inflated clock rates measured early in the outbreak seem to represent a systematic bias in the estimates, and not simply high variance (i.e., low precision) resulting from a small dataset.

### 3.3 Variability in the evolutionary rate ratio dN/dS

Although the molecular clock rate tells us the extent to which a virus is accumulating changes in its DNA, it does not imply anything about the rate of adaptation. Instead, adaptive evolution is frequently inferred from the *dN*/*dS* rate ratio. Traditionally, a value of dN/dS>1 indicates positive selection, under the assumption that an excess of non-synonymous changes is driven by adaptation. (However, on the whole-gene level, one is unlikely to observe dN/dS>1 even under positive selection, because purifying selection across the entire gene tends to dominate the overall estimate to produce dN/dS<1.) At short timescales, there are relatively few observed sequence differences, and selection has not yet had sufficient time to purge slightly deleterious mutations from the population. As a result, sequence data from early in emerging outbreaks can either bias the *dN*/*dS* rate ratio (Rocha et al. 2006; Kryazhimskiy and Plotkin 2008; Mugal, Wolf, and Kaj 2014) or make the *dN*/*dS* estimate unreliable.

**Figure 2.**
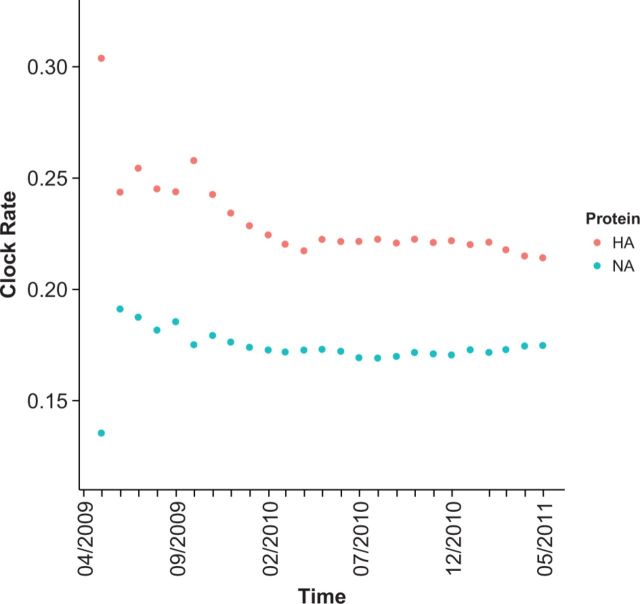
Whole-gene *dN*/*dS* estimates for pH1 hemagglutinin and pN1 neuraminidase. Each point represents the average *dN*/*dS* at the specified time point for the pH1 (red points) and pN1 (blue points) genes. All of the *dN*/*dS* values were calculated by maximum likelihood using HyPhy (Kosakovsky Pond, Frost, and Muse 2005). For each gene, the first month yielded unpredictable, inaccurate *dN*/*dS* estimates, but estimates from the data were systematically elevated between months 2–8 for pH1 and 2–11 for pN1. After approximately 11 months for pH1 and 8 months for pN1, the mean *dN*/*dS* value largely converged to the long term estimate obtained after 25 months

We inferred whole-gene *dN*/*dS* values for each monthly aggregate of data (Fig. 2). For both pH1 and pN1, our estimates that included only the first month of data were highly inaccurate, where pH1 was substantially overestimated and pN1 was substantially underestimated. After this first month, variability in monthly estimates leveled out, but, as had been the case for the molecular clock, estimated *dN*/*dS* values continued to decrease over time, until stabilizing after approximately 12 months. This trend suggests that early estimates are inaccurate and biased upwards with respect to long-term averages. In general, even though short divergence times do tend to produce elevated *dN*/*dS* values, exceedingly short time frames of 1 month may yield largely imprecise estimates, due to lack of accumulated variation (see also next subsection).

### 3.4 Distribution of site-wise dN/dS

**Figure 3.**
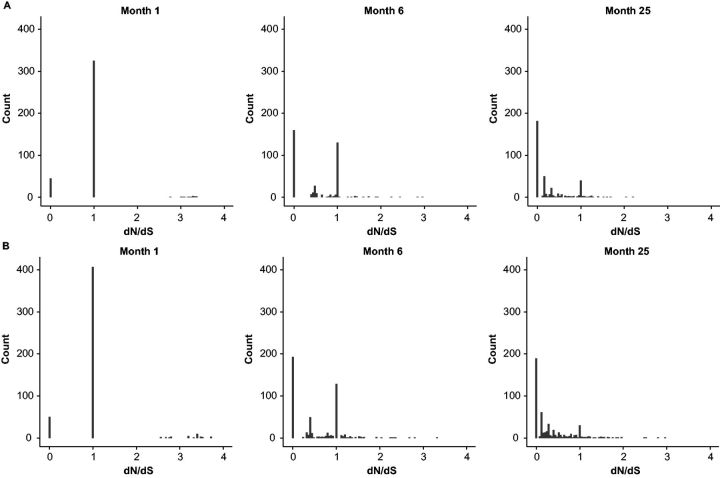
Distribution of site-wise *dN*/*dS* for pH1 hemagglutinin and pN1 neuraminidase. *dN*/*dS* distributions containing aggregated data from the 1st, 6th, and 25th month are shown for pH1 in panel A and for pN1 in panel B. The first month of data featured a majority of sites with dN/dS=1. Most of these sites were uninformative sites that had not experienced any mutations. The maximum likelihood inference approach sets *dN*/*dS* to the arbitrary value of 1 for these sites. After 6 months, roughly half of the sites were informative, although half still showed dN/dS=1. Finally, after 25 months of divergence, the majority of sites had informative *dN*/*dS* values. Distributions for all months are shown in Supplementary Figures S3 and S4

We next analyzed how sample divergence time influenced site-specific *dN*/*dS* estimates for pH1 and pN1. In Fig. 3, we show how the sample divergence time influenced the overall distribution of site-wise *dN*/*dS* estimates for pH1 (Fig. 3A) and pN1 (Fig. 3B). Both genes displayed the same general trend. With only a single month of data, the *dN*/*dS* distribution was unimodal, centered around dN/dS=1. As we considered increasingly longer time windows, the mode around dN/dS=1 decayed, while a second mode near dN/dS=0 arose and took up an increasingly larger percentage of the data. At 25 months, the mode around dN/dS=1 had almost completely disappeared and the distribution consisted almost entirely of the mode near dN/dS=0. By comparison, the distribution of site-wise *dN*/*dS* for all human H3 hemagglutinin sequences from 1991 to 2004 showed a completely unimodal distribution centered near dN/dS=0 (Supplementary Fig. S1). Thus, the site-wise *dN*/*dS* distribution had after 25 months largely, but not completely, reached its final shape.

**Figure 4.**
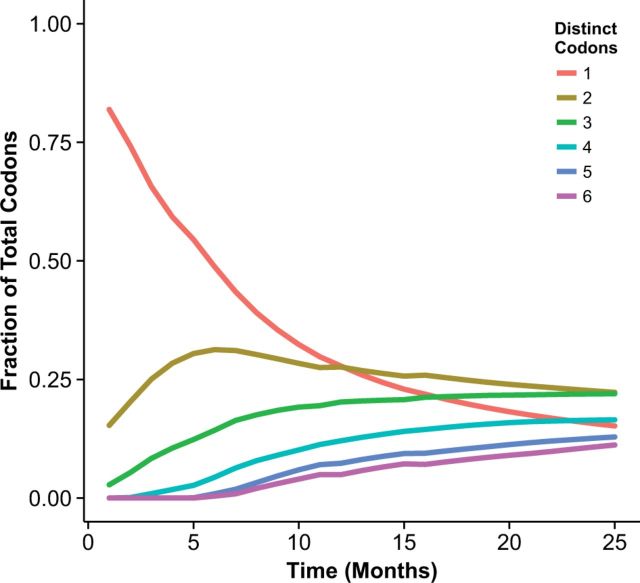
Fraction of alignment columns with distinct numbers of codons, plotted over time for hemagglutinin. Alignment columns with 1 distinct codon are completely conserved, while columns with two, three, etc. distinct codons have experienced at least one, two, etc. mutations. At 6 months, approximately half of all sites had not yet experienced a mutation, and even after 25 months, 16 out of 503 sites in pH1 remained completely conserved

We investigated the origin of the mode at dN/dS=1 and found that it could generally be traced to completely conserved codon sites in the alignment. If a site has experienced neither a synonymous nor a non-synonymous mutation, both *dN* and *dS* are undefined. In this case, the maximum-likelihood inference algorithm outputs the arbitrary starting value for *dN*/*dS* of 1. More generally, for site-wise *dN*/*dS* estimates to be reliable, the sequence alignment needs to be sufficiently diverged at each codon site. We estimated overall divergence by counting the fraction of codon sites in each alignment that had 1, 2, 3, etc. distinct codons, and plotted these fractions over time (Fig. 4 and Supplementary Fig. S2). The fraction of sites with one distinct codon corresponds to the sites that have not experienced any mutations, while the fractions of sites with two, three, etc. distinct codons correspond to the sites that have undergone at least one, two, etc. mutations. We found that the mode at *dN*/*dS* declined in proportion to the number of completely conserved sites. At 5–6 months, approximately 50 per cent of all sites were still completely conserved in both proteins (Fig. 4 and Supplementary Fig. S2), and even after 25 months, 16 out of 503 sites in hemagglutinin and 14 out of 387 sites in neuraminidase still did not show any evidence of evolutionary divergence.

**Figure 5.**
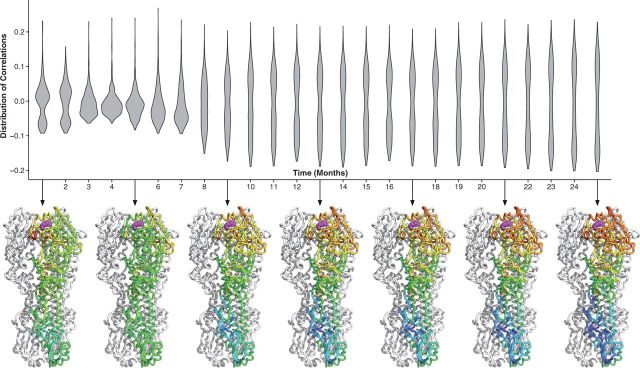
Temporal development of geometric evolutionary constraints in hemagglutinin. The violin plots show the distribution of *dN*/*dS*–proximity correlations using each possible site in the hemagglutinin protein as the reference point. The violin plot should be viewed as a horizontal histogram; thus, the wider the violin plot, the higher the number of reference sites with that correlation. Underneath the violin plots, we map these correlations onto the protein structure at 4-month time intervals. The hemagglutinin protein (PDB ID 1RD8) is shown in its native trimer structure, but the correlations are plotted onto just one of the monomers. The correlation pattern stabilizes after approximately 8 months of divergence

**Figure 6.**
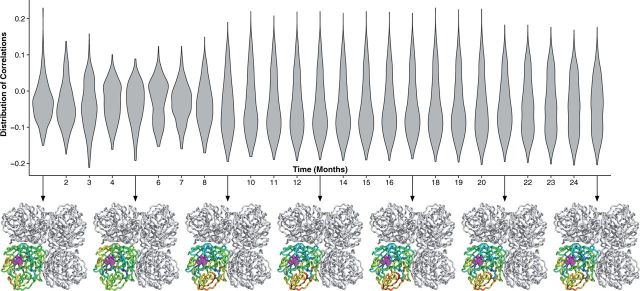
Temporal development of geometric evolutionary constraints in neuraminidase. The violin plots show the distribution of *dN*/*dS*–proximity correlations using each possible site in the neuraminidase protein as the reference point. The violin plot should be viewed as a horizontal histogram; thus, the wider the violin plot, the higher the number of reference sites with that correlation. Underneath the violin plots, we map these correlations onto the protein structure at 4-month time intervals. The neuraminidase protein (PDB ID 3TI3) is shown in its native tetramer structure, but the correlations are plotted onto just one of the monomers. The correlation pattern stabilizes after approximately 8 months of divergence

### 3.5 Effect of limited divergence on inferring important regions of hemagglutinin and neuraminidase

We have previously shown that proximity to the sialic acid-binding site in H3 hemagglutinin is one of the two best predictors (the other being relative solvent accessibility [RSA]) of site-wise *dN*/*dS* (Meyer and Wilke 2015). More specifically, if we calculate correlations between site-wise *dN*/*dS* and the inverse distance to a reference site, then reference sites near the sialic-acid binding region yield significant positive correlations while reference sites away from the sialic-acid binding region yield either no correlation or negative correlations. Thus, sites closer to the sialic-acid binding region tend to have higher *dN*/*dS*. By mapping these correlations onto the protein structure, we can visualize this result as a heat map that shows the sialic acid-binding region as the hottest region, and sites progressively farther away from this reference are proportionately colder (Meyer and Wilke 2015).

Assuming sufficient divergence for accurate site-wise estimates of *dN*/*dS*, we expected to find a similar trend in both pH1 hemagglutinin and pN1 neuraminidase. In contrast to the receptor binding protein hemagglutinin, however, neuraminidase is an enzyme with a functionally constrained chemical active site. Therefore, we expected neuraminidase to display the opposite trend from that of hemagglutinin: sites closer to the active site of neuraminidase should appear colder, that is, more evolutionarily conserved.

We found for both proteins that these expected trends emerged with sufficient evolutionary divergence. For pH1, just as for H3, sites closer to the sialic acid-binding site appeared hotter on our maps (Fig. 5). Similarly, for pN1, sites nearer the catalytic core were colder than the exposed surface (Fig. 6). Moreover, we found that these trends stabilized after approximately 8–10 months for both proteins. The temporal development of these geometric evolutionary constraints is also shown in Supplementary Videos S1 and S2.

## 4 Discussion

Molecular evolutionary metrics, namely the molecular clock rate (nucleotide substitution rate) and the *dN*/*dS* evolutionary rate ratio, are widely used to infer the evolutionary dynamics of viruses. With the uptake of rapid sequencing technologies, such evolutionary estimates have found widespread use in tracking emerging epidemics. However, the underlying models used to infer these metrics make a key assumption that genetic variation arises solely from fixed differences among sequences, and the influence of segregating mutations is ignored. Theoretical work has demonstrated that this assumption produces biased or inaccurate clock rate and *dN*/*dS* estimates at small divergence times (Ho et al. 2005; Rocha et al. 2006; Ho et al. 2007; Kryazhimskiy and Plotkin 2008; Peterson and Masel 2009; Ho et al. 2011; Mugal, Wolf, and Kaj 2014). Importantly, during the initial stages of an emerging infectious disease, viral sequences are separated almost exclusively by transient polymorphisms rather than fixed differences, making evolutionary estimates unreliable. It is not known, however, how much divergence time is required to obtain accurate and reliable clock rate and *dN*/*dS* estimates.

We found that reasonably accurate measurements for each metric could be obtained with 4–6 months of data for clock rate and 8 months of data for *dN*/*dS*. Moreover, we found that site-specific *dN*/*dS* values were largely uninformative during the first 6 months of the outbreak but started to become reliable for longer time windows. Our results provide empirical evidence of the theoretical predictions that short-time scales produce biased and inaccurate estimates for both the molecular clock and *dN*/*dS*. Since we obtained these results for influenza virus, which is one of the most rapidly evolving viruses, it is possible that more slowly evolving viruses will require substantially more time before estimates converge to their long-term values.

The pH1N1 influenza that swept the globe in 2009 is among the most studied pandemics in history. Indeed, one of the most widely cited early epidemic studies was performed on pH1N1 (Smith et al. 2009); in addition, there are several studies that are narrower in scope (Rambaut and Holmes 2009; Furuse et al. 2010) but have comparable results to our own. For example, one earlier study which included pH1N1 data from March 2009 to May 2009 also used the molecular clock rate and whole-gene *dN*/*dS* (Smith et al. 2009). In pH1, they found a similarly elevated whole-gene dN/dS=0.32 in the early epidemic and dN/dS=0.21 in non-outbreak swine clades. By comparison, we found nearly identical values for whole-gene *dN*/*dS* with the 1st month and 25th-fifth month of data, respectively. For neuraminidase, previous work found dN/dS=0.26 during the initial outbreak and dN/dS=0.18 for the non-outbreak swine data. In contrast, we found dN/dS=0.135 for the first month and dN/dS=0.175 for the 25th month. Thus, with the exception of the first month of data for neuraminidase, our results are in excellent agreement with prior work. It is unclear what caused the discrepancy in the first month; however, estimates are highly unreliable for these very short time scales, and minor differences in the set of selected sequences may result in very different *dN*/*dS* estimates.

In addition to the good agreement between the non-outbreak and long-divergence-time pH1N1 *dN*/*dS* estimates, we also found that our estimates of the molecular clock rate were similar to earlier work (Rambaut and Holmes 2009; Smith et al. 2009; Hedge, Lycett, and Rambaut 2013). In particular, we used essentially the same approach and data as did Hedge, Lycett, and Rambaut (2013). In that study, the authors used whole-genome data to calculate, among other things, the molecular clock rate of pH1N1. As with our analysis, the rate calculated in the first month was very different from that of the rest of the outbreak. After the second month of sampling, they found a mean clock rate of $3.93\times 10^{-3}$ subst./site/year for the entire pH1N1 genome. Likewise, Hedge, Lycett, and Rambaut (2013) found a slight decrease in clock rate and a narrowing of the HPD interval as more data was added to the estimates. For comparison, with 2 years of data, we found a molecular clock rate of $5.2\times 10^{-3}$ subst./site/year for hemagglutinin and $4.6\times 10^{-3}$ subst./site/year for neuraminidase. Thus, both hemagglutinin and neuraminidase have higher mean molecular clock rates than the average of the pH1N1 genome; this is not surprising considering the pressure these immune-exposed proteins should experience. In addition, our estimates were similar to those found earlier for the immediately pre-outbreak reference from swine of $3.67\times 10^{-3}$ subst./site/year for hemagglutinin and $3.65\times 10^{-3}$ subst./site/year for neuraminidase. With 25 months of data, our estimates for both hemagglutinin and neuraminidase were virtually identical to the whole-genome estimate of $5.02\times 10^{-3}$ subst./site/year with early pH1N1 data (Rambaut and Holmes 2009). Therefore, 25 months of data were sufficient to capture the long-term evolutionary trends.

There are at least two distinct mechanisms that contribute to inaccurate estimates of clock rates and *dN*/*dS* early in an outbreak. First, segregating polymorphisms lead to inflated estimates for both quantities. This effect has been discussed extensively in the literature (Ho et al. 2005; Rocha et al. 2006; Ho et al. 2007; Kryazhimskiy and Plotkin 2008; Peterson and Masel 2009; Ho et al. 2011; Mugal, Wolf, and Kaj 2014). Second, limited sampling divergence results in estimates with low precision. When the total number of accumulated mutations is low, it is simply not possible to obtain precise estimates of clock rates or *dN*/*dS*, regardless of whether the observed mutations are fixed substitutions or transient polymorphisms. This effect will be more pronounced for per-site estimates than for whole-gene estimates, but it can be present in either case. For the data we analyzed here, limited sampling divergence was likely a major contributing factor for the observed whole-gene estimates for the first month of data. For the per-site estimates, on the other hand, we needed 8–10 months of data to obtain reasonably precise estimates for a majority of sites.

What do our results imply for the practice of analyzing disease outbreaks as they occur? Although there is little doubt that the molecular clock rate and whole-gene *dN*/*dS* are important for understanding and quantifying emerging outbreaks, both metrics by themselves provide relatively limited information about a particular outbreak. In a given outbreak scenario, after estimating these quantities, one has to assess what these estimates mean, and in particular, whether any deviations from estimates obtained for past outbreaks reflect an actual biological difference in the current outbreak or simply a biased or imprecise estimate due to limited sampling divergence and transient polymorphisms. One possible approach towards answering this question is to proceed as we have done here, by subdividing the data into successively longer temporal intervals and assessing whether estimates seem to converge to the long-term values. However, this approach requires a large amount of sequence data collected over a substantial time window.

Alternatively, one could attempt to internally verify that a sufficient level of accumulated variation exists by investigating whether or not the data fit an expected pattern. For example, there are a number of structure-based constraints that guide protein evolution. Various studies have shown that RSA, weighted contact number, energy of mutation, and local packing density of sites can account for some portion of variation in site-wise *dN*/*dS* for diverse proteins (Meyer and Wilke 2013; Meyer, Dawson, and Wilke 2013; Huang et al. 2014; Shahmoradi et al. 2014; Sikosek and Chan 2014; Yeh et al. 2014). Moreover, in influenza specifically, it is known that proximity to the sialic acid-binding site is a strong constraint on hemagglutinin evolution (Bush et al. 1999b; Hensley et al. 2009; Koel et al. 2013; Meyer and Wilke 2015); in fact, a combined model with RSA and proximity to the sialic acid-binding site performs similarly to a cross-species comparison of site-wise *dN*/*dS* estimates (Meyer, Dawson, and Wilke 2013; Meyer and Wilke 2015).

We showed here that, after 25 months of divergence, the number of uninformative sites dropped to a sufficiently low number, and the resultant site-wise estimates were sufficiently accurate, to display the same proximity-based pattern as seen in H3 hemagglutinin (Supplementary Fig. S1). For pH1, that pattern started to emerge clearly in November of 2009 with 8 months of accumulated mutations. Moreover, this date could be inferred from the distribution of site-wise *dN*/*dS* estimates; it is the first month when substantially more sites are informative than are uninformative (Supplementary Fig. S3). Likewise, for pN1, we showed that a similar proximity metric can be used for the enzyme active site. However, in the case of neuraminidase, the active site was more constrained rather than less constrained, as was the case in hemagglutinin. This finding makes sense intuitively, since enzymes must retain a functional active site at all costs. Further, we found that the expected pattern began to emerge after the same 8-month interval, and again, this interval could have been inferred based on the site-wise *dN*/*dS* distribution (Supplementary Fig. S4). Therefore, we have found that by inspecting the change in the distribution of site-wise *dN*/*dS* estimates over time, we could identify the time point at which site-wise estimates became reliable.

## Supplementary data

Supplementary data is available at *VEVOLU Journal* online.

Supplementary Fig. S5
