# Supplementary material for: Time dependence of evolutionary metrics during the 2009 pandemic influenza virus outbreak
Source: Virus Evol. 2015 Aug 24;1(1):vev006. doi: 10.1093/ve/vev006 (PMC4710376; doi:10.1093/ve/vev006)
Supplement: Supplementary Fig. S5 [file ve_vev006_index.html]

Supplementary Data | Virus Evolution

## Supplementary Data

files

- Supplementary Data - mov file
- Supplementary Data - mov file
- Supplementary Data - pdf file
