# Supplementary material for: Time dependence of evolutionary metrics during the 2009 pandemic influenza virus outbreak
Source: Virus Evol. 2015 Aug 24;1(1):vev006. doi: 10.1093/ve/vev006 (PMC4710376; doi:10.1093/ve/vev006)
Supplement: Supplementary Fig. S5 [file Supplementary_Data.pdf]

Supporting Information

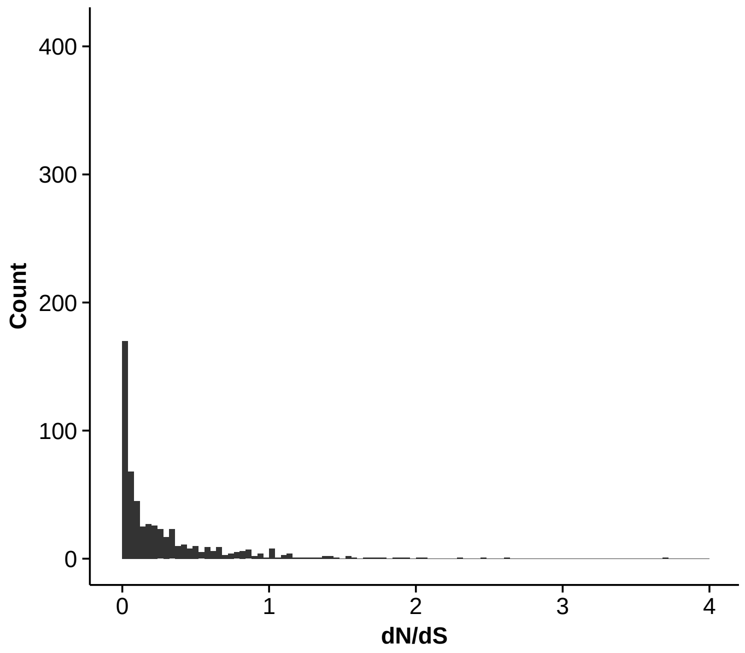

Figure S1: Distribution of site-wise dN/dS for all H3N2 hemagglutinin sequences from 1991-2014. The figure shows the distribution of site-specific dN/dS estimates including from over 20 seasons of H3 hemagglutinin. Unlike the dN/dS distributions for pH1N1, the H3 dN/dS distribution reflects that sequences have sufficiently diverged such that nearly all sites have accumulated some mutations. Therefore, there are relatively few uninformative sites with $dN/dS = 1$ compared to pH1 and pN1 distributions with a full 25 months of data.

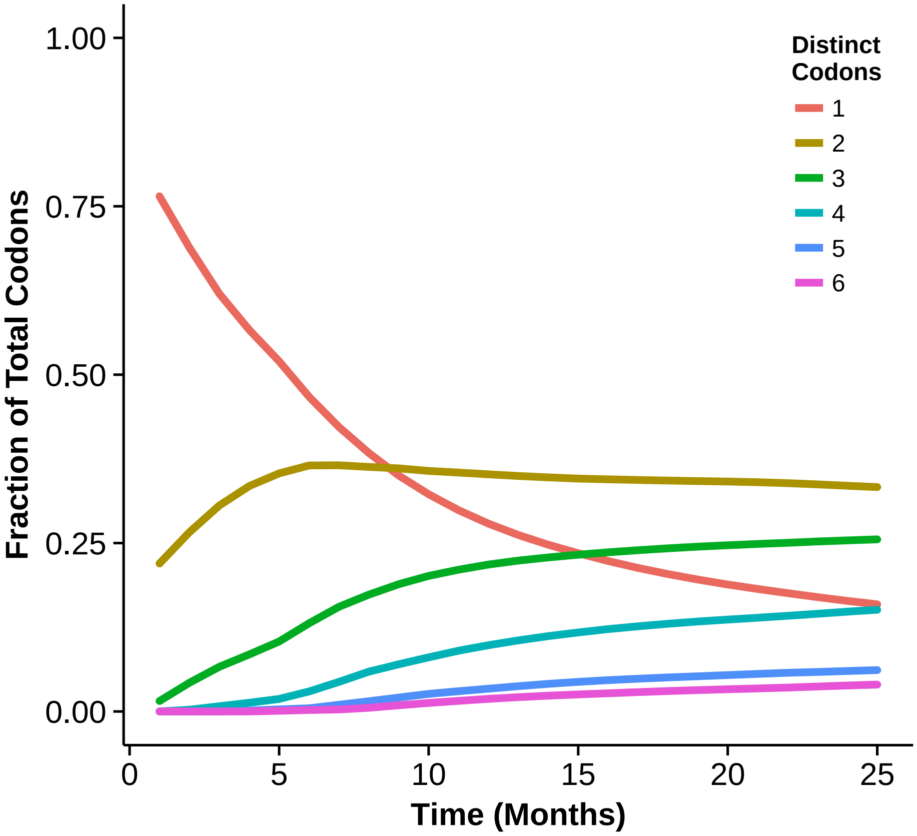

Figure S2: Fraction of alignment columns with distinct numbers of codons, plotted over time for neuraminidase. Alignment columns with 1 distinct codon are completely conserved, while columns with 2, 3, etc. distinct codons have experienced at least 1, 2, etc. mutations. At five months, approximately half of all sites had not yet experienced a mutation, and even after 25 months, 14 out of 387 sites remained completely conserved.

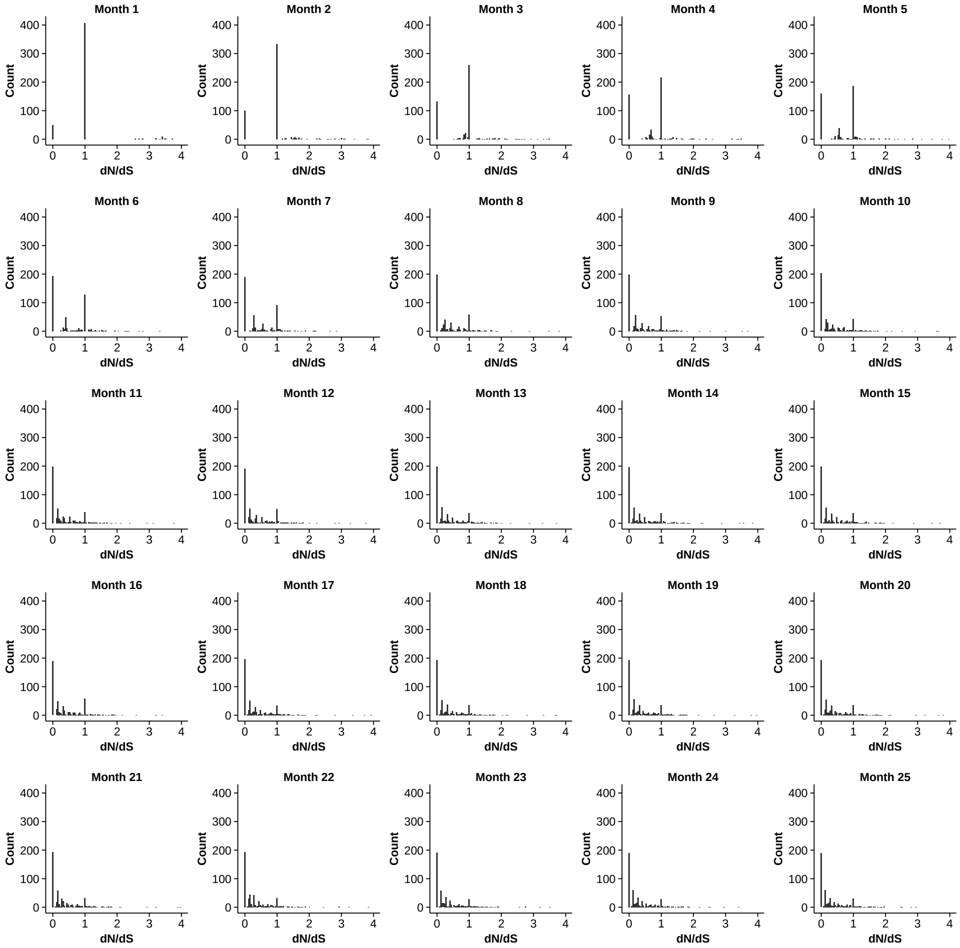

Figure S3: Distribution of site-wise dN/dS values for pH1 hemagglutinin for increasingly longer time windows.

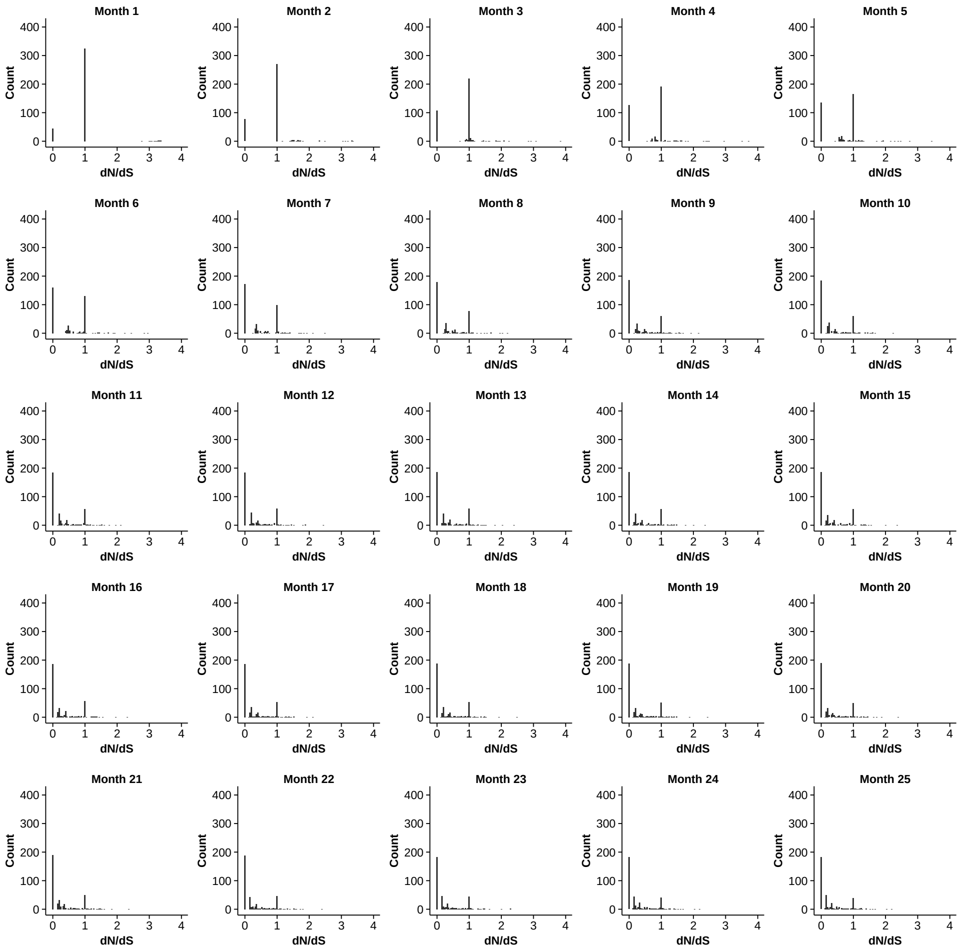

Figure S4: Distribution of site-wise dN/dS values for pN1 neuraminidase for increasingly longer time windows.

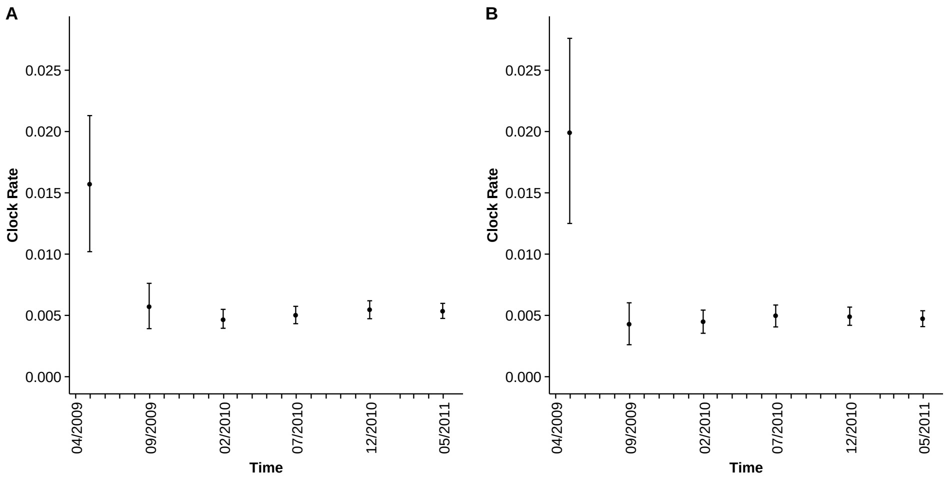

Figure S5: Repeat of the clock rate calculations using heterogeneous sites model with four gamma categories. In panel A, we show the molecular clock rate over time for pH1, and in panel B we show the molecular clock rate over time for pN1. The error bars represent the HPD 95% of the mean, as reported by BEAST. The plot shows a four-fold decline in the substitution rate estimates from single month of data to 25 months of aggregated data. Further, the molecular clock HPD 95% for the first 2 months of data, for both pH1 and pN1, does not overlap the final clock rate, indicating that these early estimates are in no way representative of the long-term estimates.

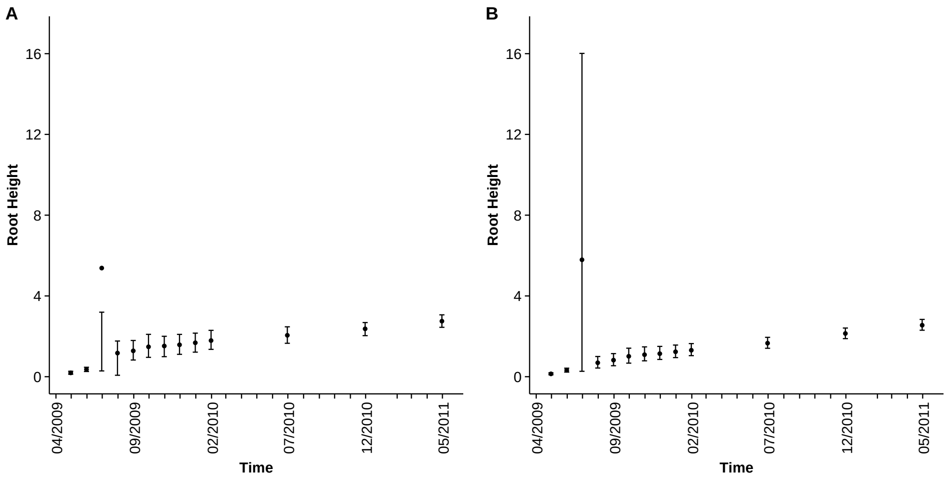

Figure S6: Root height versus time during the 2009 outbreak. In A, we show the plot for pH1. In B, we show the plot for pN1. The interval is the HPD 95%. The root height shows an increase in sequence sample divergence as the outbreak progresses. The aberrant value in the third month is the result of necessary downsampling started during that month of data. Nevertheless, after the fourth month, the root height continues with the appropriate trajectory.

S1 Video

Strength of binding positive selection identification in hemagglutinin. The video shows the strength of correlation between the set of distances from each reference C_α to each site in the protein and site-wise dN/dS. Hemagglutinin is shown in the functional trimeric form that it takes *in vivo*. Sialic acid (the hemagglutinin receptor) is highlighted in magenta. If a site in the protein is red, distances from that reference point were a strong, positive predictor of dN/dS; thus, red regions were under relatively strong diversifying selection. Alternatively, if a site in the protein is blue, distances from that reference point were a strong, negative predictor; thus, the blue regions were under relatively strong purifying selection. Finally, if a site in a protein is green, distances from that site were not predictive of dN/dS. The movie shows that for several months after pH1N1's emergence, mutations had not sufficiently accumulated for important protein regions to be reliably inferred. After a year, however, predictions stabilized, and the sialic acid-binding region showed evidence of strong, positive selection.

S2 Video

Strength of binding positive selection identification in neuraminidase. The video shows the strength of correlation between distances from each reference $\alpha-$Carbon to each site in the protein and dN/dS at the same sites. Neuraminidase is shown are the function tetrameric form it takes *in vivo*. The neuraminidase inhibitor zanamivir is highlighted in magenta. If a site in the protein is red, distances from that reference point were a strong, positive predictor of dN/dS; thus, red regions were under relatively strong diversifying selection. Alternatively, if a site in the protein is blue, distances from that reference point were a strong, negative predictor; thus, the blue regions were under relatively strong purifying selection. Finally, if a site in a protein is green, distances from that site were not predictive of dN/dS. As with hemagglutinin, after about a year, predictions stabilized, with the catalytic site showing evidence of strong negative selection and the external surface showing evidence of weak positive selection.
